# Donkey-assisted therapy in mental health conditions: a systematic review

**DOI:** 10.3389/fpsyt.2025.1680983

**Published:** 2025-10-16

**Authors:** Alessia Fulgenzi, Antonio Raffa, Carmela De Domenico, Marcella Di Cara, Simona Leonardi, Adriana Piccolo, Fabio Mauro Giambò, Giulia Marafioti, Angelo Alito, Giulia Leonardi, Laura Turriziani, Michele Panzera, Angelo Quartarone, Francesca Cucinotta

**Affiliations:** ^1^ IRCCS Centro Neurolesi Bonino Pulejo, Messina, Italy; ^2^ Department of Biomedical, Dental Sciences and Morphological and Functional Images, University of Messina, Messina, Italy; ^3^ Physical Rehabilitation Medicine Department, University Hospital A.O.U. “G. Martino”, Messina, Italy; ^4^ Centro Specializzato Universitario per gli InterventiAssistiti con gli Animali, Università degli Studi di Messina, Messina, Italy

**Keywords:** animal-assisted intervention, animal-assisted therapy, donkey therapy, neurodevelopmental disorders, psychiatric disorders

## Abstract

**Introduction:**

This paper evaluates the effectiveness of donkey-assisted therapy in neurodevelopmental disorders in children, adolescents, and adults.

**Methods:**

Registered in the PROSPERO database (CRD42025644921) and conducted according to Preferred Reporting Items for Systematic Reviews and Meta-Analyses, the review included studies from ScienceDirect (Elsevier), Web of Science, and PubMed (National Library of Medicine), searched up to 31 December 2024. The inclusion criteria were (a) pilot studies, randomized control studies, and uncontrolled clinical trials, (b) dealing with donkey-assisted interventions (DAI) in children, adolescents, and adults age, (c) in English, Italian, Spanish, or German languages. The exclusion criteria were: (a) clinical cases, pre-prints, letters to the editor, reviews, and systematic reviews; (b) studies involving therapies other than donkey-assisted therapy.

**Results:**

Four observational studies were included. The results suggest that donkey-assisted interventions, particularly donkey-assisted therapy, help improve emotional regulation, communication, social interaction, and self-esteem in children with neurodevelopmental disorders. Improvements in autonomy, motor coordination, and social engagement have been observed in adults.

**Conclusion:**

The heterogeneity of study methodologies, variations in sample size, assessment instruments, and intervention duration limit the generalizability of the results. Future research should focus on large-scale, well-controlled studies, standardizing protocols and outcome measures to quantify the therapeutic impact of DAI in diverse populations.

## 1 Introduction

The inclusion of animals in therapeutic activities is known as animal-assisted intervention (AAI), which includes animal-assisted therapy (AAT), animal-assisted education (AAE), and animal-assisted activity (AAA) (1). AAT is a targeted intervention in which the animal is an integral part of the therapeutic process, with specific goals and objectives tailored to each individual, while AAE is an educational intervention aimed at promoting, activating, and supporting the resources and potential for individual growth and planning, relationships, and social inclusion of people experiencing difficulties. The intervention can also be group-based and promotes the well-being of people in their living environments, particularly within institutions where the individual must demonstrate adaptability. EAA contributes to improving the person’s quality of life and strengthening their self-esteem. Behavioral re-education programs are also implemented through the mediation of pets. The AAA is an intervention with recreational and socialization purposes, through which it promotes improved quality of life and healthy human-animal interaction. In AAAs, the relationship with animals is a source of knowledge, sensory, and emotional stimuli; these activities are aimed at the individual or a group of individuals and promote the value of human-animal interaction within the community for mutual well-being (2, 3). Interactions between humans and animals suggest that many people find animals to be calming and non-judgmental sources of support, as well as facilitators of social interaction (2). Based on this hypothesis, this therapeutic approach offers new possibilities of complementary treatment through various forms of interaction with the animal (4, 5). Boris Levinson ideated AAI in 1960, and it rapidly developed worldwide, binding itself to a new concept of health and wellness (6, 7). In recent decades, the field of AAT has garnered significant attention within scientific literature exploring its efficacy and application in various therapeutic contexts. Particularly, several studies have highlighted the multifaceted benefits of including animal interactions in treatment strategies. These interventions have been shown to promote emotional (8, 9), social (10, 11), and motor (12) development in children, while improving behavioral outcomes (13) and reducing symptoms associated with various neuropsychiatric conditions. There is a growing body of literature about AAT in the treatment of neurodevelopmental disorders, including Attention Deficit Hyperactivity Disorder (ADHD) (14) and Autism Spectrum Disorder (ASD) (15). As evidence continues to grow, it is becoming clear that AAT offers a promising complement to traditional therapeutic approaches, enhancing the overall effectiveness of treatment in addressing a wide range of clinical objectives. AAT may involve different animal species (e.g., donkeys, horses, dogs, rabbits, cats) (1, 16, 17). From March 25, 2015, Donkey-assisted intervention (DAT) was authorized in Italian national guidelines (18). However, only a few studies have included donkeys in the treatment of pediatric neuropsychiatric conditions or in adults with neuro-fragilities (19, 20). Despite the paucity of evidence, these animals are employed for their empathy, calm and playful behavior, ideal for stress reduction and fostering social relationships (21). Donkeys are intelligent, independent, and friendly, making them suitable collaborators in treating individuals with varying degrees of neurodevelopmental disorders across all age groups (22, 23). The choice to employ donkeys for activity/therapeutic intervention is also supported by their ethological characteristics. Despite their size, they are not perceived as intimidating, contributing to their fearless nature (24), encouraging interaction, facilitating contact, and sharing spaces. In unfamiliar situations, donkeys tend to be caring and seek to form bonds, fulfilling their socialization needs (25). Due to these characteristics, donkeys are employed in activities that minimize dominance and subordination between the animal, therapist, and the patient. Donkeys are trained to perform a task through multiple exercises that make them true collaborators for cognitive and behavioral rehabilitation (26). Donkeys facilitate various forms of physical contact, including stroking, hugging, and lying on their backs. This approach fosters a deep interaction that induces relaxation and allows individuals to experience the animal’s warmth and the rhythmic breathing (27). The interactions and contact between the donkey and the patient establish a circular relationship based on trust and the possibility of sharing emotions, fundamental to treatment. As a result, donkeys could be successfully involved in building the motivational process in the psycho-affective and psycho-cognitive areas (25). This review examines the literature data regarding DAI in pediatric and adult populations, with a focus on identifying the interventions described for the specific conditions they address.

## 2 Materials and methods

### 2.1 Information sources and search strategy

This systematic review was registered within the PROSPERO database (CRD42025644921) and was conducted according to Preferred Reporting Items for Systematic Reviews and Meta-Analyses (PRISMA) (28). ScienceDirect (Elsevier), Web of Science, and PubMed (National Library of Medicine) were searched, up to December 2024. The string search included multiple terms “Assisted Therapy” OR “Assisted Intervention” AND “Donkey”, covering all types of treatment or activity involving donkeys. In addition, the reference lists of included studies were screened to ensure that a comprehensive list of relevant articles was considered for inclusion.

### 2.2 Eligibility criteria

We included only studies that met the following inclusion criteria: (a) pilot studies, randomized control studies (RCTs) both parallel or crossover studies, uncontrolled clinical trials and observational studies; (b) dealing with donkeys AAT; (c) in children, adolescent and adult population; (d) studies in English, Italian, Spanish, German languages or other languages with available translation. The exclusion criteria were: (a) clinical cases, pre-prints, grey literature, letters to the editor, reviews, and systematic reviews; (b) studies with AAT other than donkey-assisted; (c) studies in other languages.

### 2.3 Selection process

Two blinded authors (AF, AR) searched the databases according to the inclusion criteria, removed duplicates, and then identified references were screened for title and abstracts. Thereafter, full-text articles were evaluated for eligibility by title, abstract, full text, and the specificity of the topic. Whenever ratings were discordant, a third investigator (FC) analyzed the result and reached a consensus. When overlaps were found, the largest study was included. Two authors (AF, AR) blindly analyzed the extracted data independently. After titles and abstracts were screened and duplicates removed, full-text articles were evaluated for eligibility. First, the authors coded the included studies for sample and setting characteristics. Subsequently, two authors (CDD, SL) extracted data using standard data extraction forms on details of study design, intervention, and outcomes.

### 2.4 Data extraction and evaluation

To illustrate and assess study characteristics, all variables were coded as categorical and well-defined by one author (FC). The coding process included both intermediate and final agreement checks to ensure consistency and reliability. After the initial coding, two authors (AF and AR) independently reviewed and conformed to these metrics and reached a full agreement on the coding/synthesis of the articles, starting the extraction process independently. Any discrepancies identified during intermediate checks were discussed and resolved through consensus among the authors, ensuring alignment before proceeding. At the final stage, a thorough review confirmed that full agreement had been achieved on the coding and synthesis of the articles. The data extraction processes included: a) general information comprising first author, year of publication, country, and study design; b) sample characteristics including sample size, gender distribution, mean, standard deviation and range of age; c) clinical diagnostic assessment performed by questionnaires and standardized tests, diagnostic pathological condition, numerosity of each experimental group (when applicable); d) intervention characteristics including aims and main effects, type and professional figure who carried out the intervention, number, duration and frequency of sessions, information regarding the setting of experimental intervention, and outcome measures.

### 2.5 Strategy for data synthesis

It was not possible to calculate an average effect size because of the high heterogeneity in outcomes and outcome measures. Further subanalyses were performed according to (1) sample characteristics, (2) intervention type, (3) outcome measures, and (4) synthesis of main effects.

### 2.6 Quality assessment

The Newcastle-Ottawa Scale (NOS) was employed to evaluate the quality of non-randomized studies included in the review. The NOS assesses eight domains of bias, with each domain assigned a maximum score of one star, except for the comparability domain, which can receive up to two stars. The assessed domains include: (1) representativeness of the exposed cohort, (2) ascertainment of exposure, (3) selection of the non-exposed cohort, (4) confirmation that the outcome of interest was absent at baseline, (5) comparability of cohorts based on study design or statistical analysis, (6) assessment of outcomes, (7) adequacy of the follow-up period, and (8) completeness of cohort follow-up. Studies are scored on a scale from 0 to 9, with scores categorized as poor quality (0–3), fair quality (4–6), and good/high quality (7–9). Each study was assessed for potential bias based on the different components specified in the tool. The domains assessed for each study are shown in the corresponding table. Two independent researchers assessed the risk of bias (GL, AA), and disagreements were resolved by discussion with a third author (FC).

## 3 Results

The database search resulted in a total of n. 3019 studies. Of these, 30 studies were removed as duplicates. Of the 2,989 records screened, 2,742 were excluded because they did not meet the inclusion criteria. Exclusion reasons included: studies focusing on non-mental health contexts or interventions not involving donkeys (wrong background), papers with descriptive or theoretical designs without empirical data (wrong study design), and studies involving animals other than donkeys or populations outside the target group of adults, children, and adolescents (wrong population). A total of 247 studies were assessed for eligibility, with 243 records excluded.

**Figure 1:**
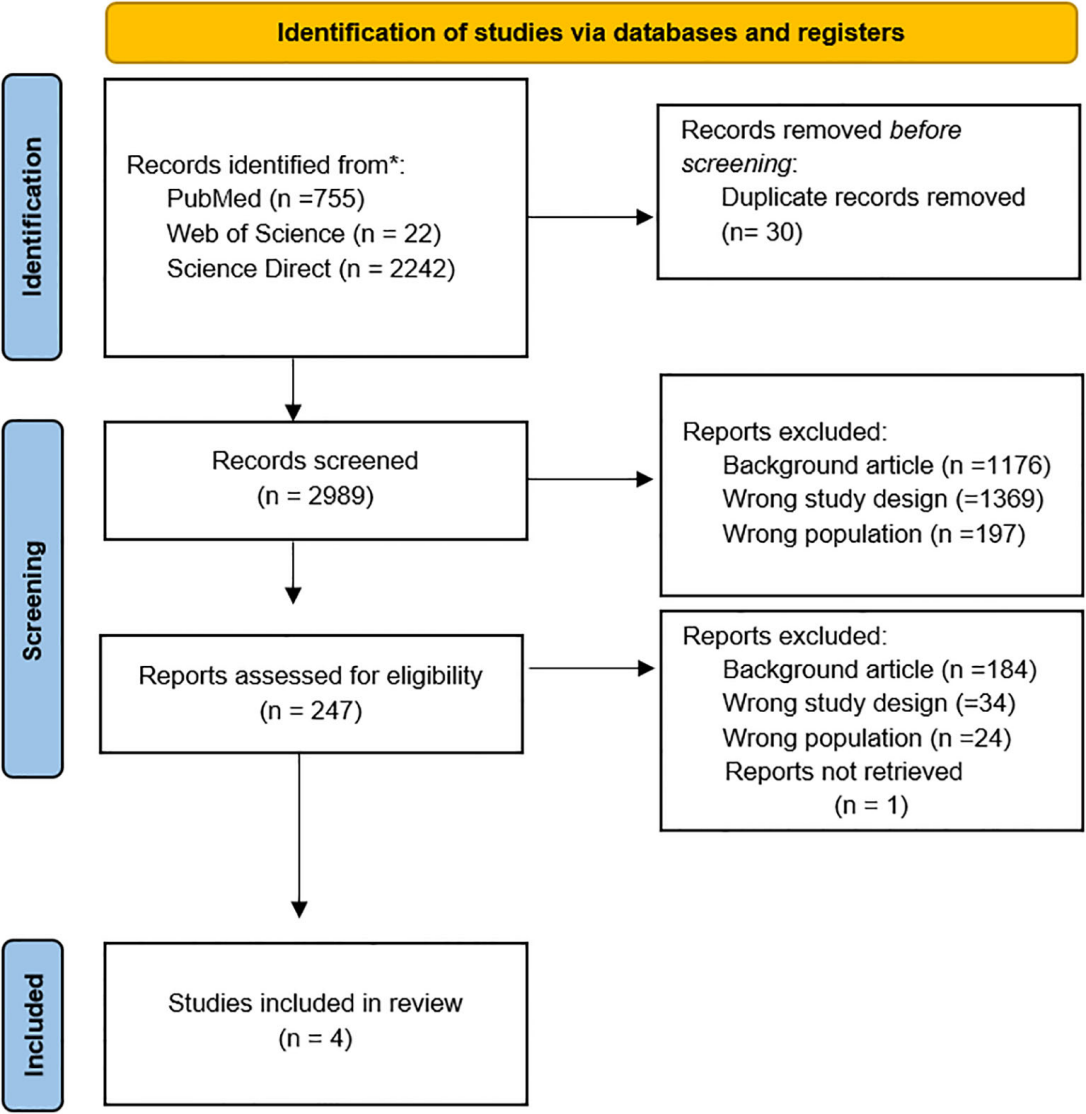
PRISMA flowchart for the literature search strategy. The figure illustrates the search and review process with the total number of articles included in this review.

All details were reported in the PRISMA flowchart (**Figure 1**). Finally, a total of 4 observational studies, conducted between 2011 and 2024 in Europe (n=3) and North America (n=1), were selected. All study characteristics are summarized in **Table 1**.

**Table 1:** Summary of study characteristics.

First author	Study title	Study design	Intervention	Intervention provide	Duration in months (frequency of sessions/week)	Aim	Outcome measure	Investigated domains	Effect of intervention	Withdrawal	Side effects
Boroni et al. (2011)	Effect of equestrian therapy and onotherapy in physical and psycho-social performances of adults with intellectual disability: a preliminary study of evaluation tools based on the ICF classification	Observational studies	DAT	Riding instructors, mental health professionals and psychologists.	12 months (NS)	Borioni applied a DAT to enhance spontaneous communication and autonomy; moreover, this study evalued timing of the improvements and persistence over time.	The A tool and the B tool, which are based on the ICF criteria	Instructors: autonomy, motor-praxis, affective-relational, cognitive; psychologists: autonomy, motor-praxis, neuro-psychological, affective-relational, cognitive, communication.	A general improvement was observed in subjects undergoing rehabilitation with the horse, for the psychological area in particular in autonomy, motor practice, affective-relational, and cognitive areas, while for instructors, in autonomy, affective-relational, and cognitive areas. For Onotherapy, on the other hand, in the psychological area, greater improvements were observed in the areas of autonomy, affective-relational, and cognitive, while for instructors in motor practice and communication.	NR	NR
Corallo et al. (29)	Improvement of self-esteem in children with specific learning disorders after donkey-assisted therapy	Observational studies	DAT + individual neuropsychological training	NR	6 months (45 min)	DAT was applied to improve the self-esteem of dyslexic children and test whether these changes had an impact on reading and writing skills.	DDE; TMA	Word reading, non-word reading, reading text, word writing, non-word writing, writing sentences with homophonic words TMA: interpersonal, competence, emotional, scholastic, familiar, corporeal.	A general improvement was observed in both word reading and text reading, specifically in the scores relating to correctness and speed, in the speed of reading non-words and in the correctness of writing words, non-words and sentences with homophonic words. In addition, an improvement was also found in interpersonal TMA scores and total TMA.	NR	NR
De Rose et al. (23)	Donkey-assisted rehabilitation program for children: a pilot stud	Observational studies	DAT	NR	6 months (45-minute session weekly)	The main objective was to improve the emotional-affective-relational status of treated children	Emotional and relational parameters, projective test	Duration of focused attention, spontaneous approach to the animal, assertiveness level, patient-therapist relationship, learning of target behaviors, use and type of verbal communication, physical expression, emotional participation, Enjoyment/Aversion to contact	Is no association between the use of verbal communication and emotional participation in our 4 patients, whereas there is an association between physical expression and spontaneous approach towards the animal.	NR	NR
Harvey et al. (30)	A Program Evaluation: Equine-Assisted Psychotherapy Outcomes for Children and Adolescents	Observational studies	EAGALA model of EAP	Mental health professional and riding instructors	10 weeks (90 min, once per week)	Improve mental health and well-being through interaction with horses or donkeys in a controlled environment	BASC-2; PRS; TRS; pattern of response index; the consistency index.	Anger, Aggression, Adaptability, Bullying, Emotional self-control, Composite externalizing and internalizing, Hyperactivity, Anxiety, Depression, Atypicality, Attention, Executive functioning, social skills, study skills.	For the group of children, parents observed positive behavior change in: anger, bullying, aggression, adaptability, emotional self-control. while Parents of the adolescent participants reported positive change in: anger, bullying, hyperactivity, anxiety, depression, atypicality, attention problems	NR	NR

ICF-CY, the International Classification of Functioning Disability and Health-Children and Youth; ID, intellectual disability; DAT, donkey assisted training; NR, Not Reported; DDE, Developmental Dyslexia Evaluation; TMA, Multidimensional Self-Esteem Test; EAGALA, Equine-Assisted Growth and Learning Association; EAP, equine-assisted psychotherapy; BASC-2, Behavioral assessment system for children; PRS, Parent Rating Scales; TRS, Teacher Rating Scales.

### 3.1 Sample characteristics

The sample includes both patients with neurodevelopmental disorders and adults with psychiatric conditions. Specifically, three studies included a sample of children, and only one study included an adult sample. The pathological conditions addressed with DAI in pediatric age were dyslexia (n.1), emotional dysregulation and behavioral problems (n.1). The last study involved a clinical population with different diagnoses (severe language and motor impairment, ADHD, Intellectual Disability (ID)). On the other hand, adult patients enrolled by Boroni et al. (19) presented mainly ID. The overall number of participants in the included studies was 70, with a range from 4 to 35 sample. The male-to-female ratio was reported in 3 of 4 studies, as one study did not provide information on gender distribution. The total number of males was 24, and the total number of females was 11. The overall gender representation ratio in the three studies with available data was M:F = 2:1. The age range of participants in the reviewed studies was 6-38.6 years (M = 17.15; SD = 12,44). The age range of the pediatric population was 6–17 years, with a mean of 10 years., while the age range of the adult population was not reported but the age mean was 38,6 y.o., ethnicity is reported in none of the reviewed studies. Clinical diagnostic assessment was reported by only two of the included studies. Specifically, one study enrolled patients with ID diagnosed by a psychiatrist and a psychologist through a physical examination and the standardized interview on adaptive skills Vineland Adaptive Behavior Scale (31). The other study involved dixlessic patients and assessed intellectual quotient with Wechsler Intelligence Scale for Children-IV (WISC-IV) (32); Developmental Dyslexia Evaluation (DDE) (33); evaluates mainly reading and writing skills in children, to identify specific difficulties related to dyslexia and Multidimensional Self-Esteem Test (TMA) (34); for the assessment of self-esteem (See **Table 2**).

**Table 2:** Patients demographics.

First author	Sample size	Age mean (sd); range (yy)	Ethnicity	Sex ratio M:F	Diagnosis	Diagnostic assessment
Boroni et al. (2011)	15	38,6 (8.6); NR	NR	13:2	ID	A physical examination and the Vineland
Corallo et al. (29)	16	9 (NR); 7-12	NR	8:8	SLD	WISC-IV; DDE; TMA.
De Rose et al. (23)	4	9 (NR); 6-12	NR	3:1	Severe language disturbance; motor impairment; emotional retardation; ADHD; mild mental retardation; low self – esteem; Down syndrome with moderate mental retardation.	NR
Harvey et al. (30)	35	12 (NR); 7 - 17	NR	NR	Neurodevelopmental disorders (i.e., emotional dysregulation and behavioral problems)	NR

ID, Intellectual Disability; Vineland, Vineland Adaptive Behavior Scales SLD, Learning Disorders; WISC-IV, Wechsler Intelligence Scale for Children-IV; DDE, Developmental Dyslexia Evaluation; TMA, Multidimensional Self-Esteem Test; NR, Not Reported; ADHD, Attention Deficit Hyperactivity Disorder.

### 3.2 Intervention type and main aims

The studies analyzed present a wide range of heterogeneous interventions, with different aims and procedures. The professionals involved in the treatment were also very different. Indeed, in two studies, interventions were delivered by a multidisciplinary team composed of riding instructors, mental health professionals, psychologists, and animal co-therapists (20, 30). Conversely, the remaining studies did not specify the qualifications of the therapists involved (23, 29). The therapeutic intervention duration ranged from 10 weeks to 12 months. All the studies employed sessions of the frequency of one meeting per week, lasting 45–60 minutes. The therapy setting was not specified in any study. No withdrawal or side effects were reported in the analyzed studies; however, no specific measures for safety data collection were reported. The goals of the therapies reported in the studies were mainly divided into emotional and cognitive domains. Specifically, the therapeutic goals pursued were varied, with interventions aimed at increasing self-esteem, improving language skills, enhancing attention and autonomy, and improving in the emotional-social domain. Borioni (19) applied a DAT to enhance spontaneous communication and autonomy; moreover, this study evaluated the timing of the improvements and persistence over time. His program consisted of three phases: (1) approach and contact, (2) interaction with the donkey, and (3) teaching the donkey to respond to commands. In Corallo’s study (29), DAT was applied to improve self-esteem in dyslexic children and verify whether these changes had an impact on reading and writing skills. The program was composed of several stages. It began with an initial introduction to the animal’s environment and simple interaction between the children and the donkey. This phase was followed by grooming, cleaning, and physical contact activities. Then, children lead the donkey along a designated exercise trail. Afterwards, training focused on saddling, riding, and driving the donkey through specific exercises. In the final stage, children were encouraged to socialize with the donkey by greeting and hugging it. In the study by De Rose et al. (23), the main aim was to improve the emotional-affective-relational state of treated children. The intervention was structured into several phases, which can be categorized into three main domains: physical interaction with the donkey, assuming responsibility for its care, and engaging in interpersonal relationships. Harvey and colleagues (30) applied the Equine Assisted Growth and Learning Association (EAGALA) model of equine-assisted psychotherapy to enhance social behaviors of adolescents with mental health problems or family-home issues. It is designed to help individuals, families, and groups improve their mental health and well-being through interaction with horses or donkeys in a controlled environment. The EAGALA used a ten-week DAT program involving a group of peers on the areas of personal and social development. EAGALA’s experiential model, called equine-assisted psychotherapy (EAP), seeks to promote a standardized model for incorporating horses into the growth and learning environment of people engaged in psychotherapy. This therapy typically spans 10–12 weeks. The program began a period of familiarization and adaptation to the environment and the group of peers that will constitute the therapeutic community, followed by sessions on group dynamics, coping skills, active listening, teamwork, self-control, self-awareness, authenticity, and emotional safety. The program concluded with a final session designed to recognize and honor the participants’ developmental progress and achievements.

### 3.3 Outcome measures of investigated domains

The primary outcomes and domains investigated in these studies were emotional and cognitive functioning. Specifically, the studies by Borioni (20), Corallo (29), and De Rose (23) focused on examining both emotional and cognitive components. In Borioni et al. (20), the authors developed an assessment tool based on the International Classification of Functioning, Disability and Health (ICF) and the International Classification of Functioning, Disability and Health: Children & Youth version (ICF-CY). A team made up of psychiatrists, neurologists, psychologists, and equestrian instructors developed this instrument. Two tools were developed according to the therapeutic objectives: Tool A was coded by psychologists, and Tool B was coded by equestrian instructors. Tool A consisted of 60 items, divided into 4 levels, to assess: autonomy, motor praxis area, neuropsychological area and affective-relational area, mental cognitive area, and communication. The same areas were evaluated by Tool B, but in only 13 comprehensive items. Corallo et al. (29) used the TMA to estimate DAT effects on general self-esteem and its components: body self-esteem, emotional, family, interpersonal, and the perception of control over events. Moreover, the authors chose to evaluate possible effects on reading and writing skills using Developmental Dyslexia Evaluation. De Rose et al. (23) investigated attention, spontaneous approach with the donkey, level of assertiveness, use and type of verbal communication, emotional participation, patient-therapist relationship, learning of target behaviors, physical expression, enjoyment of contact, and aversion to contact. These domains were evaluated using a form of three-level, from low to high level, completed by the therapist after each treatment session. The emotional-affective-relational state was further assessed through a graphical representation of the experience with the donkey, evaluated as projective production. This assessment was performed at three time-points: start of treatment, intermediate stage (3 months after the beginning), and at the end of treatment. Harvey et al. (30) investigate Anger, Aggression, Adaptability, Bullying, Emotional self-control, Composite externalizing and internalizing behaviors, Hyperactivity, Anxiety, Depression, atypicality, Attention, Executive functioning, social skills, and study skills through the Behavioral Rating System for Children (BASC-2) (35). BASC-2 is a comprehensive assessment tool used to evaluate behavioral and emotional functioning. It utilizes multiple perspectives to gain a holistic understanding of the individual’s behavior in various settings. It includes five core components, of which only 2 were used by the authors: Parent Rating Scales (PRS) and Teacher Rating Scales (TRS), tailored to different age groups and settings. Specifically, the questionnaire utilizes a mixed item-response format, as some of the items require a true/false answer, whereas others require an assessment on a 4-point scale of behaviors frequency. Finally, the BASC-2 generates profiles of obtained scores, and two response validity indicators, a pattern response index and a consistency index, also useful to indicate respectively, the extent of the change and to detect inconsistency of the responses.

### 3.4 Synthesis of main effects

The analysis of the included studies suggests that DAT can lead to significant improvements in affective-relational domains, including social skills and non-verbal communication, along with enhanced capacity to interact with the external environment. These findings are consistently associated with a general enhancement in the patients’ self-esteem, particularly in the pediatric population. Secondary outcomes, reported in a limited number of studies, include gains in autonomy, better anger management, improved adaptability, and enhanced emotional self-regulation. Specifically, Borioni and colleagues (20) in their observational study, examined adults with intellectual, emotional, communicative, and psychomotor disabilities. The authors found a relative improvement in individuals’ skills, with a major effect on autonomy level, affective-relational, and cognitive areas, for those who undergo equine-assisted therapy; however, DAT was implemented only on a smaller number of subjects, with a similar trend to the other group but a smaller number of statistically significant results. About the timing of the improvements and the persistence over time of obtained results, the authors revealed that the majority of subjects reached their maximum improvement after 3 months of therapy in almost all areas, except for cognitive performance, reached after 6 months. The four patients with neurodevelopmental disorders treated by De Rose et al. (23) showed a significant improvement in non-verbal communication skills during treatment. The majority of patients showed a better spontaneous approach to the donkeys, and among this group, two patients exhibited an improved level of verbal communication. These results suggest that the presence of the donkey in the therapeutic setting may favor the modulation of verbal language and the maintenance of stable body expression, facilitating contact with the external environment and improving the child’s self-esteem. The results obtained by Corallo et al. (29) confirm that onotherapy combined with traditional therapy can help improve the school performance and self-esteem of children with dyslexia, compared to an as-usual intervention. Specifically, DAT led to an improvement in interpersonal self-esteem values, in other words, the subject’s assessment of their perceived social relationship with others. These significant improvements were not present in the control group. Finally, the study of Harvey et al. reported a moderate effect of the equine-assisted psychotherapy efficacy as a therapeutic approach on emotion management. Specifically, parents of children noted changes in anger management, adaptability, aggressive behaviors, and greater emotional self-control. This was consistent with observations from parents of adolescents, who also reported improvements in levels of anxiety, depression, hyperactivity, and attention problems. The teachers involved reported a smaller change than the parents; however, significant in anxiety, social skills, and composite internalizing behaviors among children. Moreover, in the adolescent group were reported significant change mainly in the hyperactivity scale.

### 3.5 Quality assessment

A total of four observational studies published between 2011 and 2024 were included in this systematic review. These studies evaluated the effectiveness of donkey-assisted therapy on the physical and psychosocial functioning of both adults and children with neurodevelopmental disorders. Based on the quality assessment using the Newcastle–Ottawa Scale (NOS), three studies were rated as “poor quality,” while only one study was rated as “fair quality”. The representativeness of the exposed cohort was clearly defined in three studies (23, 29, 36), and the exposure assessment, based on objective data and structured interviews, was adequately described in three studies (20, 23, 29). Regarding the selection of unexposed cohorts, only one study (29) clearly stated the exclusion criteria applied to the sample. Outcome assessment for variables not present at baseline was thoroughly reported in two studies (20, 23). However, no studies reported adequate follow-up of the cohorts, and only one study (20) had a follow-up duration long enough (12 months) to allow for the observation of outcomes. Additionally, the comparability of cohorts, based on study design or statistical adjustment for relevant prognostic factors, was inadequate in three studies, with only one study (20) addressing this domain appropriately. Overall, the included studies showed a high risk of bias. **Table 3** summarizes the risk of bias across different domains for each study. There was strong inter-rater agreement between the two reviewers (GL and AA) who independently conducted the quality assessments.

**Table 3:** Quality assessment of the included studies by using the Newcastle-Ottawa Scale (NOS) tools for the non-randomized trials.

Publication	Exposed representation	Ascertainment of exposure	Selection of the non-exposed	Outcome was not present at start of study	Comparability of cohorts	Assessment of outcome	Sufficient follow-up time	Adequacy of follow-up of cohorts	Total
Borioni et al., 2012	0	1	0	1	1	1	1	0	5
Corallo et al., 2023	1	1	1	0	0	0	0	0	3
De Rose et al., 2011	1	1	0	0	0	1	0	0	3
Harvey et al., 2017	1	0	0	0	0	0	0	0	1

## 4 Discussion

This systematic review aimed to investigate the current scientific evidence on DAT. To the best of our knowledge, this is the first systematic review evaluating interventions and effects, without age or pathology limits. The results highlight the therapeutic potential of DAI in individuals with neurodevelopmental and psychiatric disorders. However, the number of studies in the literature is relatively small (only four), and several aspects still need to be investigated to produce evidence-based results on effectiveness. Most of the studies investigated the utility of DAT in a pediatric population. Only one study reported on an adult sample, limiting the generalization of the results to this specific population. Furthermore, the heterogeneous nature of the pathologies under study made it difficult to identify specific therapeutic targets. However, most patients shared deficits in social skills and emotional regulation; this, together with the results shown, suggests the usefulness of DAT in patients with emotional and social impairment (36). From a methodological point of view, clinical diagnostic assessment was reported by only two of the included studies, suggesting the need for more attention in the inclusion/exclusion criteria and for the diagnostic phase and design of future studies (37, 38). Overall, the analyzed studies report a wide range of heterogeneous interventions with different therapeutic aims and procedures. However, most studies reported the same structured phases of DAT, from initial interaction to cooperative engagement. This progressive adaptation and therapeutic alliance building are crucial for patients with emotional and cognitive vulnerabilities (39), and could be an asset in the programming of these interventions. Most studies evaluated the usefulness of DAT alone, while only one study integrating DAT with conventional neuropsychological treatments (e.g., cognitive-behavioral therapy). Further studies need to be conducted to verify whether the combination of these approaches can improve treatment outcomes, particularly in the cognitive domains (29, 40). The outcome measures used appear to focus on two levels: 1) the patient’s behavior during the interventions; 2) the generalization of the learned behavior. Specifically, tracking behavior data in each session may be used to evaluate attention, spontaneous approach with the animal, use and type of verbal and non-verbal communication, and emotional participation (23). On the other hand, the use of standardized reporting tools by both the clinician (20) and the parent (30), together with direct neuropsychological tests on the patient (29), can provide a complete and comprehensive idea of the effectiveness of the intervention. There are still too few studies on DAT in the literature, so it seems useful, from an exploratory and scientific perspective, to include both types of measures in the design of future studies. The findings suggest that DAI, particularly DAT, contributes to improvements in affective-relational domains, specifically in terms of how social relationships and social skills are perceived, including verbal and non-verbal communication. There is also an increased ability to engage with the external environment. The analyzed studies demonstrate that DAT may support the improvement of non-verbal communication, emotional expression, and self-esteem in children with neurodevelopmental disorders, including ADHD, dyslexia, and language impairments. In the adult population, improvements were observed in autonomy, motor coordination, and social engagement, reinforcing the role of donkey-assisted therapy in broader rehabilitation strategies. These benefits are consistent across various age groups and clinical populations, supporting the inclusion of DAT as a complementary therapeutic approach. The unique behavioral characteristics of donkeys—their calm temperament, predictability, and responsiveness- appear to cultivate a safe and engaging environment for therapeutic interaction, setting them apart from other animal-assisted interventions (24, 25, 27). Interaction with donkeys could therefore prove useful in improving autonomy, emotional well-being, and communication skills in patients with developmental disorders and cognitive deficits, but further research with specific methodological considerations is needed. Indeed, despite promising results, this review revealed some methodological concerns. Most studies had small sample sizes (range 4–35 participants), with a significant male prevalence, limiting the generalizability of the findings. Only one study reports intervention on the adult population. There are still too few studies on DAT in the literature, so it seems useful from an exploratory and scientific point of view to better define the outcome measures to be included. Indeed, it seems useful to include in the design of future studies both patients’ behavior assessment during interventions, as well as specific neuropsychological tests and standardized clinician and caregiver treatment opinions. Additionally, the duration of treatment varied considerably (from 2.5 to 12 months), which made it difficult to assess the long-term effects of the therapy comparatively.

Another issue concerns the experimental design of the studies: three were classified as “poor quality” according to the NOS scale, with a high risk of bias due to the lack of control groups, inadequate follow-up, and unclear selection criteria. Only one study (20) achieved a “fair” quality rating, emphasizing the need to improve research methodology in this field (25). Furthermore, future studies should explore whether combining DAT with conventional therapy yields greater effectiveness compared to DAT administered as a standalone intervention. Finally, there is also a lack of, randomized controlled studies, making it difficult to establish strong causal inferences regarding the efficacy of DAT. A common weakness of all the analyzed studies is the absence of evaluation measures for the animals involved, as well as a complete disregard for their fundamental characteristics. Given that the human-animal relationship is a valuable element in the context of IAA, it is crucial to guarantee the well-being of the animal in the therapeutic setting. Therefore, future studies must consider not only the inclusion/exclusion criteria for the human sample, but also for the animal sample. Furthermore, they should include measures of animal welfare during therapeutic sessions to assess the effects of these interventions on the animals involved. The fundamental value of AAI is represented by the absolute importance of animal welfare in the settings that must be appropriately monitored (41). The high proportion of studies with poor methodological quality highlights significant risks of bias, particularly in areas such as the selection of non-exposed cohorts, outcome assessment, comparability between groups, and adequacy of follow-up. This overall low level of methodological rigor substantially limits the reliability of the findings and precludes the drawing of clear or robust conclusions. To establish more definitive evidence, further well-designed observational studies and RCTs are needed. Future research should focus on larger-scale, well-controlled studies, standardizing protocols and outcome measures to better quantify the therapeutic impact of DAT across populations differing in age and pathology. While the findings are promising, further research is essential to refine intervention protocols, establish standardized guidelines, and explore the long-term benefits of this innovative therapeutic approach. The future priority should be to implement high-quality methodological studies to validate the benefits of this intervention and effectively integrate it into existing therapeutic protocols (26).
